# Asylum Workers' Association

**Published:** 1914-05-30

**Authors:** 


### Asylum Workers' Association.

The Secretary announced at the annual meeting—a full report of the speeches at which appeared in our columns last week—that the following awards had been made for long and meritorious service: Gold Medals: Mr. A. A. Williams, Hanwell Mental Hospital; Miss J. Towns, Glasgow Royal Mental Hospital. Silver Medals: Mr. J. Walters, Oxford County Mental Hospital; Miss A. Cornaby, Holloway Sanatorium. Bronze Medals: Mr. J. Jones, Glamorgan County Mental Hospital; Mr. J. Duke, Notts County Mental Hospital; Mr. J. J. Kehoe, Cork District Mental Hospital; Miss A. Munro, Glasgow District Mental Hospital. In one or two instances the recipients were present, and were handed their medals by the President.

